# Assessing the Accuracy and Reliability of Cone-Beam Computed Tomography (CBCT) in Diagnosing Grade II and III Furcation Involvement Compared to Traditional Clinical Examination Methods

**DOI:** 10.7759/cureus.55117

**Published:** 2024-02-28

**Authors:** Renad A Alotaibi, Rehab Abdulaziz, Nancy Bery, Muneer A Alotaibi, Shaul Hameed Kolarkodi

**Affiliations:** 1 Medicine, College of Dentistry, Qassim University, Buraydah, SAU; 2 Diagnostic Radiology, King Fahad Specialist Hospital, Buraydah, SAU; 3 Periodontology, College of Dentistry, Qassim University, Buraydah, SAU; 4 General Dentistry, Buraydah Dental Clinic, Buraydah, SAU; 5 Maxillofacial Surgery and Diagnostic Sciences, Qassim University, Buraydah, SAU

**Keywords:** mesio-palatal (mp), disto-palate [dp], clinical attachment level [cal], periodontal pocket depth [ppd], computerized tomography [ct], cone-beam computed tomography [cbct], furcation involvement [fi]

## Abstract

Introduction

Chronic periodontal diseases can lead to bone defects and tooth loss, making accurate diagnosis essential for treatment. Various methods are used for diagnosing furcation involvement, with clinical examination and cone-beam computed tomography (CBCT) being the most effective. CBCT produces reliable images with submillimeter resolution, revealing marginal bone contours and furcation defects. Limited studies show that CBCT is more precise than clinical detection for diagnosing furcation involvement. Therefore, CBCT technology can be considered as an adjunct method for furcation involvement detection. This study tests the accuracy and efficiency of CBCT and clinical examination in detecting furcation involvement.

Material and methods

The study included 25 patients (68 molars) with generalized periodontitis of stage II to IV, Grade B and C. Inclusion criteria required at least two intrabony defects with probing depth > 6mm in both jaws and criteria of periodontitis in at least 30% of the teeth present.

Results

The study compared clinical examination and CBCT readings for measuring maxillary and mandibular teeth furcation involvement. The results show significant differences between clinical and CBCT measurements of maxillary teeth furcation involvement, particularly in specific areas and types of furcation involvement. In contrast, there were significant differences between clinical and CBCT readings for only a few measurements of mandibular teeth furcation involvement. Therefore, the study suggests that CBCT imaging may be beneficial for accurate diagnosis and treatment planning in cases of furcation involvement in maxillary teeth.

Conclusion

CBCT imaging is a reliable adjunct method for detecting furcation involvement in maxillary teeth, while clinical examination alone may not be sufficient. Therefore, the use of CBCT technology should be considered in cases where accurate detection of furcation involvement is necessary for successful treatment outcomes. However, further studies with larger sample sizes are needed to confirm these findings.

## Introduction

Chronic periodontal diseases are inflammatory conditions affecting the periodontium and are among the most common oral diseases affecting adults. Molars have an increased susceptibility to develop periodontal diseases, leading to various complications, such as attachment loss and alveolar bone resorption, and as the alveolar bone gradually loses its structure, bone defects become apparent around the teeth and in the interradicular (furcation) region, ultimately resulting in tooth loss. Periodontal disease can lead to multiple complications [1]; therefore, an accurate diagnosis is essential for determining prognosis and strategizing an active treatment plan. Many different methods are used for diagnosing furcation involvement (FI), including evaluating probing pocket depth, clinical attachment levels, probing the furcation entrance, and periapical radiographs [2]. Digital periapical images are commonly used to obtain two-dimensional (2D) images. However, these images are insufficient to assess FI because of overlapping anatomical structures and a lack of three-dimensional (3D) information. Clinically, FI is determined using a combination of clinical examination using Nabers probe and cone-beam computed tomography (CBCT) radiographic analysis. Hamp's and Glickman's classification systems are used to categorize FI based on horizontal bone loss at the furcation area [3,4]. While the accuracy of clinical detection largely depends on an operator's skills, which can differ from one operator to another, the measurements are often based on the penetration depth into inflamed connective tissues rather than the actual depth of the interradicular bony defect [5]. Several factors such as tooth condition, inclination, root morphology, root trunk length, degree of root separation, and configuration of residual interradicular bone also affect the reliability of clinical furcation measurements [6,7]. CBCT is a technique used to evaluate the shapes of the head and neck. In contrast to traditional computed tomography (CT), CBCT produces accurate and reliable images with submillimeter resolution in all spatial dimensions at a fraction of the cost and absorbed dose required for traditional CT [8,9]. CBCT has been increasingly used in dentistry, including periodontology [10,11]. Its predictable capability to reveal marginal bone contours and infrabony and furcation defects [12] aids in the detection and treatment planning of FI in molars. To date, only a few studies have compared the diagnostic accuracy of clinical and CBCT for detecting FI [11,13,14]. CBCT is more precise in diagnosing such conditions and could be considered as an adjunct method for detecting FI. The aim of this study was to investigate and compare the accuracy and efficiency of CBCT and clinical examinations for detecting FI.

## Materials and methods

**Figure 1:**
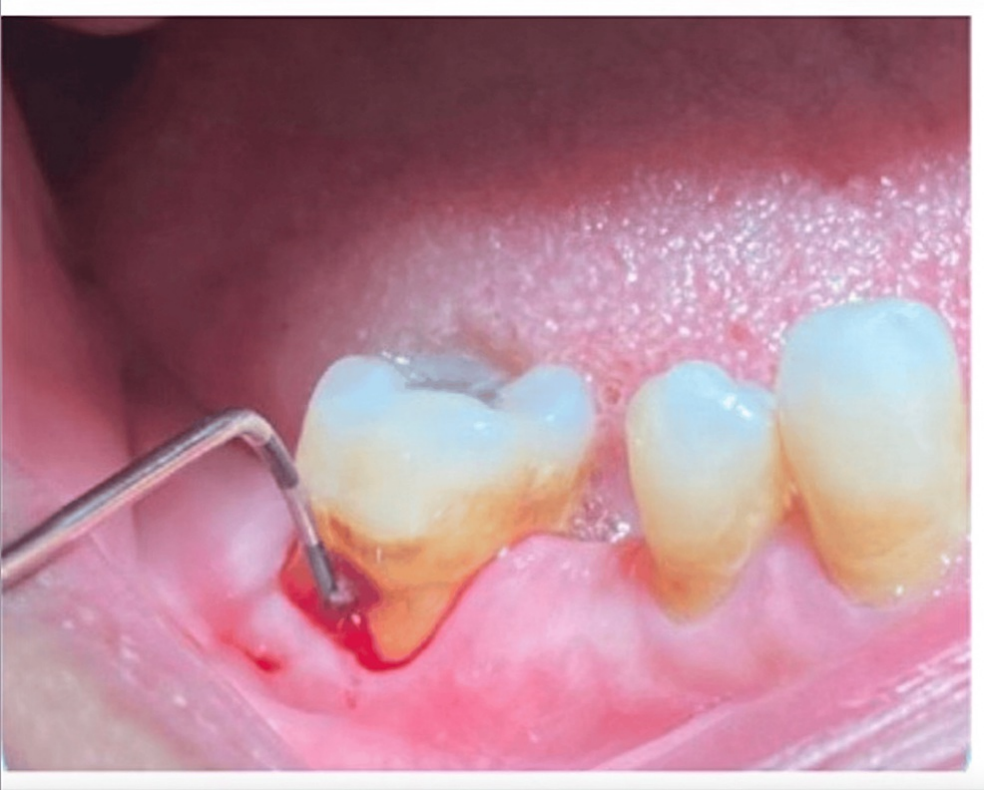
Clinical picture of grade II furcation involvement using Nabers probe

**Figure 2:**
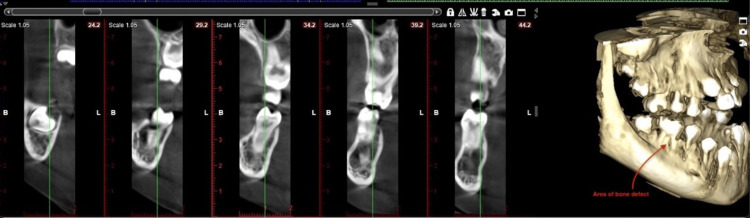
Cone-beam computed tomography image of grade II furcation involvement

**Figure 3:**
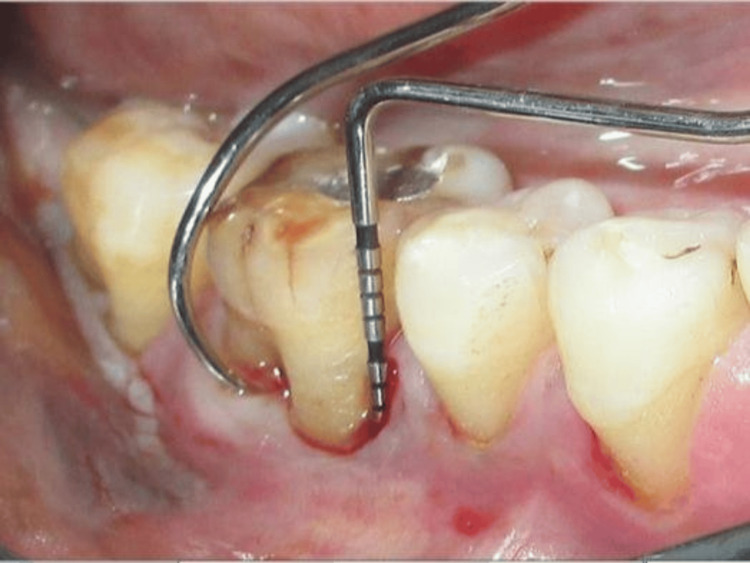
Clinical picture of grade III furcation involvement using Nabers probe

**Figure 4:**
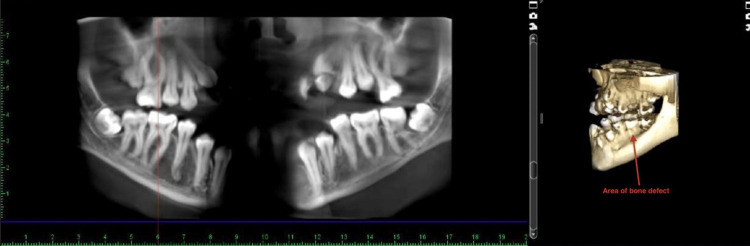
Cone-beam computed tomography image of grade III furcation involvement

Twenty-five male and female patients (68 molars with FI) diagnosed with generalized periodontitis stages II-IV, grades B and C, were selected from those visiting the outpatient dental clinic at Qassim University. The study was approved by the ethics committee of Qassim University (IRB number: EA/6095/2021). According to Glickman (1958), periodontitis is graded as grade A (slow rate of progression), grade B (moderate rate of progression), or grade C (rapid rate of progression). Additionally, periodontitis is categorized into stages as follows: stage 1 (initial periodontitis), stage 2 (moderate periodontitis), stage 3 (severe periodontitis with the potential for tooth loss). The inclusion criteria were the presence of at least two intrabony defects, with a probing depth of >6 mm in both maxillary and mandibular molars, and periodontitis in at least 30% of the teeth present (generalized periodontitis). Patients with systemic diseases, lactating or pregnant women, and those with furcation affected by caries were excluded from the study. All the patients provided written informed consent before participating in the study. The patients underwent a comprehensive periodontal examination, including the measurement of pocket depth and assessment of molar FI using Nabers probe according to the modified Glickman’s classification. A Nabers probe is a modified Marquis probe with a curved working end to access the furcation, as opposed to the straight tip of the Marquis probe. It is used to determine the extent of FI in multi-rooted teeth. Glickman’s classification of FI includes grade I, pocket formation into the furcation but intact interradicular bone; grade II, loss of interradicular bone and pocket formation but not extending through to the opposite side; and grade III, through-and-through lesion. After the clinical evaluation of FI, CBCT scans were obtained to visualize trabecular bone resorption in both sagittal and axial views. Using an axial slice, the depth of bone loss was determined by measuring the distance between the lines that intersected the neighboring root surfaces from the deepest point (Figures 1-4).

Descriptive and inferential statistical analyses of the data were performed. Since the data consisted mainly of continuous variables, including measurements of FI based on clinical and CBCT examination, means and standard deviations were calculated and tabulated for descriptive statistics.

A paired-sample test was conducted to determine and interpret any significant differences between the clinical and CBCT measurements. The mean differences between the two measurements along with the standard deviation of the mean difference, t-statistics, and p-values were tabulated and reported. Statistical significance was established at a p-value of 0.05 or less with a 95% confidence interval. All statistical analyses were performed using IBM SPSS Statistics software, version 27.0.1 (IBM Corp., Armonk, NY, USA).

## Results

We compared measurements of FI in maxillary and mandibular teeth obtained through clinical and CBCT examinations (Table 1 and Table 2). The results demonstrated significant differences between the clinical and CBCT measurements of FI in maxillary teeth, particularly in specific regions and types of FI. Significant differences were observed in the buccal region of Grade II 1st molars (p=0.002), mesio-palatal region of Grade II 1st molars (p=0.013), disto-palatal region of both Grade II 1st and 2nd molars (p=0.008 and p=0.021, respectively), buccal region of Grade III 1st molars (p=0.027), both regions of Grade III 2nd molars (p<0.001 for buccal and p=0.009 for mesio-palatal), and disto-palatal region of Grade III 1st molars (p=0.038). Therefore, clinicians should consider using CBCT imaging for accurate diagnosis and treatment planning for FI.

**Table 1:** Comparison of Clinical versus CBCT Measurements of Furcation Involvement in Maxillary Teeth CBCT: Cone-beam computed tomography

				Mean Clinical Measurements	Mean CBCT Measurements	Mean Difference	Std. Deviation of Mean Difference	t	P value^t^
Grade II furcation involvement									
Buccal		1^st^ Molar (N=14)		7.08	5.55	1.53	1.38	4.000	0.002*
		2^nd^ Molar (N=15)		7.00	6.06	-0.98	1.21	-2.915	0.091
Mesio-palatal		1^st^ Molar (N=14)		4.23	5.21	-0.84	0.95	-3.203	0.013*
		2^nd^ Molar (N=15)		4.50	4.92	0.94	1.92	1.826	0.171
Disto-palatal		1^st^ Molar (N=14)		4.46	5.30	-0.42	1.09	-1.448	0.008*
		2^nd^ Molar (N=15)		4.50	5.67	-1.17	1.67	-2.615	0.021*
Grade III furcation involvement									
Buccal	1^st^ Molar (N=14)			7.39	6.37	1.02	1.54	2.493	0.027*
	2^nd^ Molar (N=14)			6.93	5.52	-1.03	1.26	-3.074	<0.001*
Mesio-palatal	1^st^ Molar (N=14)			4.43	5.46	-0.70	1.14	-2.306	0.009*
	2^nd^ Molar (N=14)			6.50	5.95	1.41	1.24	4.259	0.315
Disto-palatal	1^st^ Molar (N=14)			4.43	5.13	0.56	1.99	1.045	0.038*
	2^nd^ Molar (N=14)			5.00	6.66	-1.66	0.87	-7.125	<0.001*
All measurements are in mm ^t^Paired sample t-test *p<0.05, significant									

**Table 2:** Comparison of Clinical versus CBCT Measurements of Furcation Involvement in Mandibular Teeth CBCT: Cone-beam computed tomography

		Mean Clinical Measurements	Mean CBCT Measurements	Mean Difference	Std. Deviation of Mean Difference	t	P-value^t^
Grade II furcation involvement							
Buccal	1^st^ Molar (N=9)	6.89	4.56	2.33	1.19	5.860	<0.001*
	2^nd^ Molar (N=3)	6.67	6.83	0.71	0.40	5.269	0.742
Lingual	1^st^ Molar (N=9)	4.67	3.96	-0.17	0.76	-0.378	<0.001*
	2^nd^ Molar (N=3)	7.00	5.50	1.50	0.87	3.000	0.095
Grade III furcation involvement							
Buccal	1^st^ Molar (N=8)	7.38	9.14	-1.76	2.27	-2.191	0.065
	2^nd^ Molar (N=6)	6.97	5.94	-1.50	1.38	-3.066	0.018*
Lingual	1^st^ Molar (N=8)	5.13	6.63	1.03	0.73	3.454	0.018*
	2^nd^ Molar (N=6)	6.00	6.09	-0.09	1.26	-.165	0.876
All measurements are in mm ^t^Paired sample t-test *p<0.05, significant							

For grade II FI in the buccal region of the first molar, the mean clinical measurement was significantly higher than the mean CBCT measurement. However, this difference was not statistically significant. For grade II FI in the mesio-palatal (MP) region of the first molar, the mean clinical measurement was significantly lower than the mean CBCT measurement. For grade III FI in the disto-palatal (DP) region of the first molar, the mean clinical measurement was significantly lower than the mean CBCT measurement.

To summarize, we observed significant differences between the clinical and CBCT measurements of grade II FI in the buccal region of the first molar and grade III FI in the buccal region of the second molar and lingual region of the first molar. These results suggest that CBCT may be more accurate in detecting FI in certain regions. However, further research is needed to confirm these findings.

## Discussion

Our study aimed to compare measurements of FI in maxillary and mandibular teeth obtained through clinical and CBCT examinations. The findings of the present study are consistent with those of previous studies comparing the accuracy of clinical and radiographic examination techniques for assessing FI. A study conducted by Alsakr et al. (2022) demonstrated that CBCT is more accurate than clinical examination in detecting FI in maxillary molars [1]. Similarly, Dommy et al. (2017) found that CBCT is more accurate than clinical examination for assessing FI in mandibular molars [2].

The results of the present study indicated significant differences between the mean clinical and CBCT measurements in certain regions of grade II FI in the first and second molars. In the buccal region of the first molar, the mean clinical measurement was significantly higher than the mean CBCT measurement, whereas in the buccal region of the second molar, the mean clinical measurement was higher than the mean CBCT measurement, but the difference was not statistically significant. This suggests that the CBCT measurements may underestimate the extent of FI, particularly in the buccal region of the first molar.

Conversely, for grade II FI in the MP and DP regions of the first molar, the mean clinical measurements were significantly lower than the mean CBCT measurements. This suggests that clinical measurements may underestimate the extent of FI, particularly in the MP and DP regions of the first molar.

These findings are consistent with those of some previous studies that have also reported discrepancies between clinical and radiographic measurements of FI [3,4]. However, it should be noted that the current study focused on a specific type of radiographic examination through CBCT, whereas previous studies used different types of radiographic measurements, such as periapical and panoramic radiographs.

The results of our study suggest significant differences between the clinical and CBCT measurements of grade III FI in the buccal, MP, and DP regions of the first and second molars.

In the buccal region of the first molar, the clinical measurements were significantly higher than the CBCT measurements, suggesting that clinicians may overestimate the severity of FI in this region based on clinical examinations alone. In contrast, in the second molar, the clinical measurements were significantly lower than the CBCT measurements, indicating that clinicians may have underestimated the severity of FI in this region.

In the MP region of the first molar, the clinical measurements were significantly lower than the CBCT measurements, suggesting that clinicians may underestimate the severity of FI in this region based on clinical examinations alone. Conversely, in the second molar, the clinical measurement was significantly higher than the CBCT measurement, indicating that clinicians may overestimate the severity of FI in this region [5].

In the DP region of the first molar, the clinical measurements were significantly lower than the CBCT measurements, suggesting that clinicians may underestimate the severity of FI in this region based on clinical examination alone [6]. Furthermore, in the second molar, the clinical measurements were significantly lower than the CBCT measurements, indicating that clinicians may have underestimated the severity of FI in this region.

The results of our study are consistent with those of previous studies suggesting discrepancies between clinical and radiographic measurements of FI [7,8]. These discrepancies may be due to limitations in clinical examinations, such as inadequate access or visibility, and in radiographic imaging, such as distortion or superimposition of structures. It is important for clinicians to be aware of these limitations and use multiple diagnostic tools, including clinical examinations and radiographic imaging, to accurately assess the severity of FI and develop appropriate treatment plans.

The results of this study indicate significant differences between the clinical and CBCT measurements of FI in mandibular teeth, specifically for grade II FI in the buccal region of the first molar. The mean clinical measurements were significantly higher than the mean CBCT measurements, with a mean difference of 2.33 mm. This finding suggests that the clinical measurements may overestimate the extent of FI in this region.

In contrast, no significant difference between clinical and CBCT measurements was observed for grade II FI in the lingual region of the first molar. The mean clinical measurement was slightly higher than the mean CBCT measurement; however, the difference was not statistically significant. This suggests that the clinical and CBCT measurements may be comparable in this region.

It is worth noting that the sample size for mandibular measurements was relatively smaller than that for maxillary measurements, which may limit the generalizability of the findings. Additionally, the study only included measurements of grades II and III FI in the mandibular molars; therefore, it is unclear whether the results can be generalized to other teeth or other types of periodontal measurements.

Various studies have compared clinical and radiographic measurements of periodontal disease [9]. A systematic review by Shaker et al. (2021) demonstrated that clinical measurements tend to overestimate the extent of periodontal disease compared to radiographic measurements [10]. However, the studies included in the review varied in terms of the type of radiographic measurement used and the type of periodontal disease being evaluated.

These results indicate significant differences between the clinical and CBCT measurements for grade III FI in the buccal region of the second molar and grade III FI in the lingual region of the first molar. In contrast, no significant differences were observed between the clinical and CBCT measurements for grade III FI in the buccal region of the first molar or lingual region of the second molar.

The findings of this study are consistent with those of previous studies that have shown that CBCT can serve as a useful tool for diagnosing FI [11,12]. In particular, measurements obtained through CBCT have been shown to be more accurate in detecting FI than that of clinical examination, especially in cases where the furcation is located in a difficult-to-access region or in areas with significant bone loss [11].

However, it is important to note that CBCT should not be used as a replacement for clinical examination [13], as clinical examination can provide valuable information about other aspects of periodontal health, such as gingival inflammation and tooth mobility. Additionally, CBCT scans should be weighed against the risks associated with radiation exposure [14].

This study has several limitations, one of which is its cost. As an observational study prone to bias, its internal validity may be affected by differences in measurement bias between examiners; hence, future studies could assess both inter- and intra-examiner validity and reliability. An additional drawback of this study is the lack of randomization. Future studies should be designed with more samples and detailed examinations to address these limitations, including exploring CBCT measurements for both horizontal and vertical bone loss.

## Conclusions

Based on our research, CBCT is primarily used for diagnosing periodontal disease, followed by its application in patient outcome studies. Infrabony defects and FI can be accurately diagnosed using CBCT. Furthermore, it provides accurate results for assessing the effectiveness of periodontal surgery and regenerative therapy. Clear guidelines and evidence for the usefulness of CBCT must be established through a larger cohort of studies that can measure diagnostic efficacy, therapeutic outcomes, and societal benefits.
